# A computational approach to demonstrate the control of gene expression via chromosomal access in colorectal cancer

**DOI:** 10.21203/rs.3.rs-2981903/v1

**Published:** 2023-06-01

**Authors:** Caleb J. Pecka, Ishwor Thapa, Amar Singh, Dhundy Bastola

**Affiliations:** 1 College of IS&T, University of Nebraska at Omaha, Omaha, NE, United States of America; 2 Department of Biochemistry and Molecular Biology, University of Nebraska Medical Center, Omaha, NE, United States of America

**Keywords:** colorectal cancer, epigenetics, chromatin accessibility, snakemake

## Abstract

Improved technologies for chromatin accessibility sequencing such as ATAC-seq have increased our understanding of gene regulation mechanisms, particularly in disease conditions such as cancer. This study introduces a computational tool that quantifies and establishes connections between chromatin accessibility, transcription factor binding, transcription factor mutations, and gene expression using publicly available colorectal cancer data. The tool has been packaged using a workflow management system to allow biologists and researchers to reproduce the results of this study. Through the application of this pipeline, we present compelling evidence linking chromatin accessibility to gene expression, with particular emphasis on SNP mutations and the accessibility of transcription factor genes. Furthermore, we have identified significant upregulation of key transcription factor interactions in colon cancer patients, including the apoptotic regulation facilitated by E2F1, MYC, and MYCN, as well as activation of the BCL-2 protein family facilitated by TP73. The code for this project is openly available on GitHub at the following address: https://github.com/CalebPecka/ATAC-Seq-Pipeline/.

## Introduction

The regulatory mechanisms of gene expression play a critical role in cell differentiation and development, especially in disease conditions such as cancer. Transcription factors (TFs) have been shown to direct the regulation of genes by recognizing transcription factor binding sites (TFBSs) to initiate transcription of downstream genes [1]. TFs are incapable of initiating transcription if their binding site is condensed around a histone octamer structure called a nucleosome. Proteins in the cell can more easily interact with uncondensed chromatin, otherwise called accessible regions of DNA. In this paper, our goal was to design and develop a computational tool that demonstrated the interactions between chromatin access, TF binding, TF mutations, and gene expression using publicly available colorectal cancer (CRC) data.

Chromatin accessibility assessment can be accomplished using a variety of protocols including DNase-seq [2], and ATAC-seq [3]. ChIP-seq [4], a protocol that analyzes TF-DNA interactions, can require hundreds of millions of cells as input [3], while the ATAC-seq protocol only requires a standard input of 50,000 cells, making the technique appropriate for research with precious cell types, including cancer cells [3]. The development of chromatin sequencing technology has made it more feasible for researchers to incorporate chromatin accessibility with the analysis of other gene regulation mechanisms. For example, the interaction between chromatin access and transcription has improved predictive models of gene expression based on HiChIP throughput data [5].

Studies have also demonstrated that there is a correlation between chromatin accessibility and gene expression. Pearson correlations have shown that accessible chromatin of promoter regions had similar correlation patterns with gene expression for both healthy and cancerous tissues [6]. Furthermore, it has been shown that inferred TF binding interactions are capable of predicting and differentiating cell types [6].

As researchers improve our ability to assess gene regulation mechanisms, there is an increased need for computational tools that can perform integrative analysis in an accurate and streamlined manner. There is a lack of tools that can perform analysis on the results of chromatin accessibility data in a user-friendly manner. Using a functional programming approach, we designed a reproducible workflow environment that requires minimal systems administration knowledge.

Our workflow uses chromatin accessibility data from The Cancer Genome Atlas (TCGA) to predict TFBSs based on motif sequences found in accessible chromatin regions [6]. These results were validated using a database of known TF motifs from JASPAR [7] as well as gene expression profiling data. In addition to statistical validation, we have incorporated a dynamic track-based visualization system that clearly shows the interaction between chromatin accessibility, TF motif sequences, and the genes they regulate. The outputs from our workflow are designed to be compatible with common file formats used in other track-based visualization applications, including the UCSC Genome Browser. Code for this project is publicly available on GitHub (https://github.com/CalebPecka/ATAC-Seq-Pipeline/).

## Materials and methods

### Project overview and reproducibility

**Figure 1:**
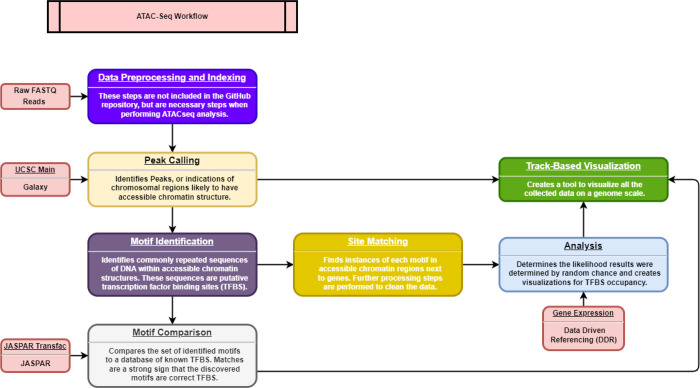
High-level overview of the project pipeline. Data from Raw FASTQ Reads, TCGA reference genome, JASPAR Transfac motifs, and Data Driven Referencing (DDR) Expression are processed by a series of scripts that lead to a Track-Based Visualization and Statistical Analysis.

**Figure 2:**
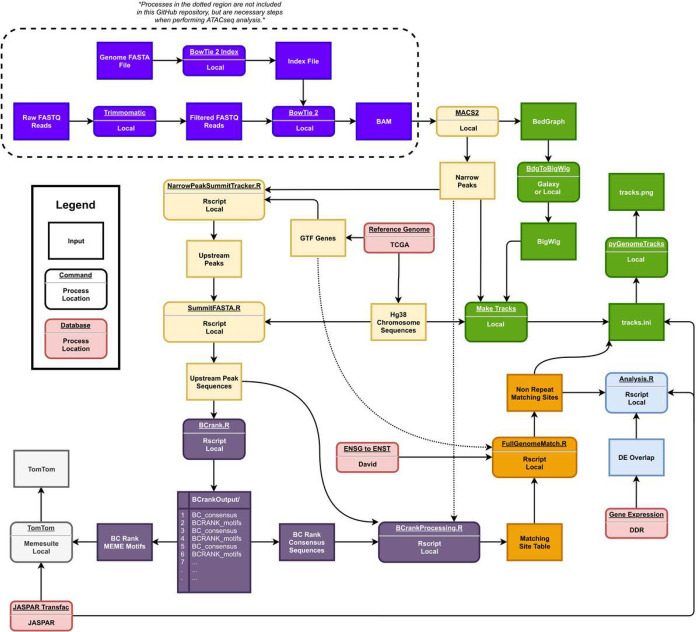
Low-level overview of the project pipeline. Inputs and commands are color-coded to reflect the high-level overview shown in Fig 1.

A high-level overview of the project can be seen in Fig 1, including Data Preprocessing, Peak Calling (to identify accessible chromatin regions), Motif Identification (to identify putative TFBSs), Motif Comparison (to compare putative TFBSs against validated databases of TFBSs), Site Matching, Statistical Analysis, and Track-Based Visualization. The high-level overview is color-coded to correspond with individual scripts, inputs, and outputs reflected in the low-level overview given in Fig 2.

Our pipeline requires the user to input a set of binary alignment map (BAM) files for analysis. All other input files are either provided in the GitHub repository or automatically installed by the pipeline. For example, the hg38 human reference genome is automatically installed in a Snakemake script. The pipeline outputs files for the genomic location of accessible chromatin regions (Upstream Peaks), motif sequences identified by BCrank (BC Rank Consensus Sequences), and layered genomic visualizations (tracks.png). For full descriptions of these files and all available fields, please refer to our GitHub documentation.

We employed Snakemake [8] to automatically detect the progress of the workflow and run necessary code based on a configuration file that can be modified by the user, making it possible for most users to ignore the technical intricacies in our low-level overview. Snakemake automatically installs conda environments required for software dependencies. We have also enabled a parameter to configure the conda environment to perfectly replicate the dependency build used in this study. The perfectly reproducible configuration may require an adaptable installation script provided with the workflow.

To the best of our knowledge, there are no other methodologies or tools currently available that can provide a meaningful comparison with our pipeline.

### Data preprocessing and indexing

ATAC-seq, RNAseq, and SNP mutation data for 41 CRC patients were preprocessed by TCGA [6]. The hg38 human reference genome was used as a reference for the Bowtie2 alignment tool [6]. Samtools sorted the mapped reads and Picard removed duplicates, resulting in a set of 41 binary alignment map (BAM) files for each of the patient samples [6]. For patients with multiple ATAC-seq BAM files, our mutation data only contained one instance of each patient ID. Seven more patients were missing mutation data in TCGA, as shown in Table 1. A supplementary file of TCGA barcodes is provided as a Table in Additional File 1. Preprocessing procedures were carried out by TCGA study and are not included in the GitHub pipeline. The pipeline requires the user to input a BAM file for each sample.

### Peak calling

MACS2 was used to identify DNA read fragment pileups, also called chromatin peaks [9]. Chromatin peaks are indicative of DNA regions where chromatin structure is accessible. Each peak contains a summit value, indicating the base-pair location where fragment pileup is highest [9]. The first step of our pipeline compiles a list of all peak summits located 100–1000 base-pairs upstream of all genes at the ENSG level.

### Motif identification and site matching

The 50 base-pair region centered around each peak was extracted as a FASTA sequence using Biostrings and the hg38 reference genome for *Homo sapiens* [10]. For each of the 41 CRC samples, BCrank created a list of 1200 motifs from the upstream FASTA sequences [11]. BCRank requires the input FASTA sequences to be ordered according to confidence level. Our script automatically sorts the sequences according to q-score, the confidence level provided by MACS2. BCrank was also used to map those motifs onto the upstream FASTA sequences in order to obtain the location of putative TFBSs [11]. We would like to note that this method is limited due to an excess of false positives, a common problem with motif prediction tools.

### Motif comparison

For each sample, the list of 1200 motifs was reformatted to be compatible with MEME Suite tools [12]. A collection of known TFs from JASPAR was converted into a similar format [7]. TomTom searched for pattern matches between the position weighted matrices of our 1200 putative TFBSs against the known JASPAR collection [13]. The FDR p-value correction method was globally employed across the TomTom results for all samples, and non-significant results were removed from the merged list of all patients (p_*adjust*_ ≤ 0.05).

### Determination of differentially expressed genes

The Data Driven Referencing (DDR) method [14] was employed to create a list of differentially expressed (DE) genes for the 31 non-duplicated TCGA CRC barcodes discussed in ”Data preprocessing and indexing”. The DDR method normalizes gene expression levels into 5 tiers based on the relative expression of housekeeping genes in each sample [14]. We chose the DDR method over other gene expression analysis tools because the DDR normalization process is better suited for accounting for non-biological variabilities [14]. Fisher’s Exact Tests were used to determine which genes are enriched in cancer samples versus healthy samples [14]. DDR outputted a list of differentially upregulated and downregulated genes after performing the FDR p-value correction method and subsetting the results (p_*adjust*_ ≤ 0.05). The resulting DE genes as well as fold changes and p-values are provided as a supplementary Table in Additional File 2.

### Creation of genome tracks

Track-based visualizations were created using pyGenomeTracks [15] [16]. Bigwig files were created to visualize chromatin accessibility using the bamCoverage program provided by deepTools [17]. Known TF motifs were collected from the JASPAR database [7]. Tracks containing known gene locations were imported from the hg38 reference genome.

## Results and discussion

### Chromatin accessibility across the CRC genome

In the Peak Calling step, MACS2 returns a score that quantifies chromatin peak accessibility by comparing the fragment pileup relative to various background regions of fragment pileup at a maximum of 10,000 base pairs away [9]. In the original TCGA study by Corces et al., the researchers noted that the MACS score was problematic due to its variability across different datasets [6]. We hypothesized that this issue may not be relevant in our study because we focused on a specific cohort of CRC patients. The experiments performed in this subsection were intended to investigate this hypothesis.

We used a one-tailed wilcoxon rank sum test to compare the mean accessibility scores across all samples in housekeeping genes versus non-housekeeping genes. Our findings revealed that the mean accessibility score for housekeeping genes is significantly higher than non-housekeeping genes (p ≤ 0.05).

In some situations, a gene will not have a mean accessibility score because MACS2 did not identify a statistically significant peak upstream of the gene in any of the 38 non-duplicated patient IDs (see Table 1). In these situations, we can quantify gene accessibility based on a second accessibility metric, whether or not a gene has an accessible upstream promoter region in each patient sample. Out of 3805 housekeeping genes [18], 128 were not identified as significant by MACS2. Our search list included 58,387 unique gene symbols, of which a total of 26,274 were not identified as significant by MACS2. Using a Fisher’s Exact Test, we concluded that the variance in chromatin accessibility can be partially explained by whether or not a gene is a housekeeping gene (p ≤ 0.05). The raw data for these statistical tests is provided as a Table in Additional File 3.

By definition, we expect housekeeping genes to be expressed in the cell at all times, as they are necessary for basic cellular functions. Therefore, we expect housekeeping genes to also have accessible promoter regions, as they constantly need to be transcribed. Our statistics support these assumptions and help to verify that the MACS2 accessibility score is a useful metric for quantifying chromatin accessibility. Therefore, in the following section (Correlation between chromatin access and gene expression), we use the MACS2 accessibility score to correlate chromatin access with gene expression. In all other cases, we quantify accessibility based on our second accessibility metric.

### Correlation between chromatin access and gene expression

For each gene, Pearson correlations were used to identify a relationship between MACS2 chromatin accessibility scores and the raw HTseq gene expression across all 38 patient samples. A two-tailed Wilcoxon rank sum test was used to compare the correlation coefficients for housekeeping and non-housekeeping genes, and we concluded that these correlations were not significantly different from each other. This specific test is not supportive evidence for the regulation of non-housekeeping genes via chromosomal access. Instead, this result supports the claim that the underlying mechanisms of chromosomal access are not specific to housekeeping or non-housekeeping genes.

Similar tests were performed to compare the correlation coefficients for CRC biomarker genes (determined using the DDR method, a tool previously developed in our lab) and non-biomarker genes [14]. A one-tailed Wilcoxon test suggests that correlation coefficients between chromatin access and gene expression are expected to be higher in our list of biomarkers acquired from DDR (p ≤ 0.05). We can interpret this result to explain the mechanisms of differential gene expression. Genes which have differential gene expression patterns can be closely tied to a respective increase or decrease in chromatin access.

### Motif comparison

The 1200 motifs produced by BCrank were a highly conservative estimate of the number of motifs necessary to describe the global pattern of TF binding in a patient. For each patient, BCrank calculated 100 motifs to describe a global optimum set. This procedure was repeated 12 times with a different seed generation each time. TomTom is a commonly used tool that identifies pattern matches between two sets of motif sequences [13]. Using TomTom, we calculated that, on average, 68% of our predicted motifs had a statistically significant pattern match with known JASPAR motifs [13].

**Figure 3:**
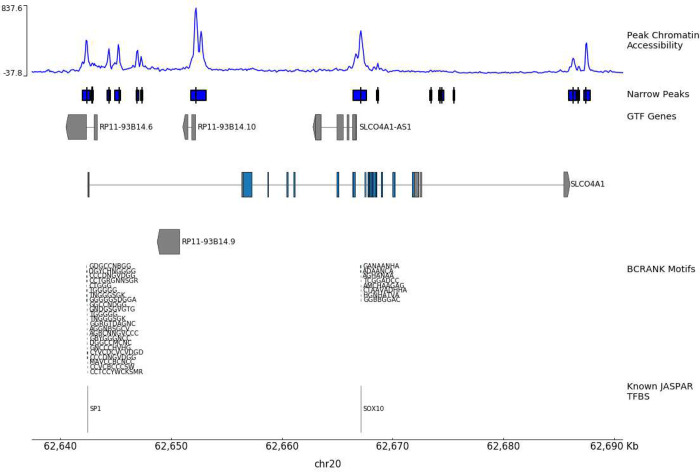
Example track-based visualization of the SLC04A1 gene. The x-axis displays the chromosomal position of our data. The top track (Peak Chromatin Accessibility) represents read fragment pileup as a method of quantifying chromatin accessibility. The blue box track beneath it (Narrow Peaks) represents MACS2’s interpretation of peak regions, as well as each peak’s summit location (black tick marks in each box). The GTF genes track shows the full reference genome labeling, as well as a small directional arrow to showcase whether a gene is transcribed on the positive or negative strand. The BCRANK Motifs track showcases our predicted motifs mapped onto the reference genome. The Known JASPAR TFBS track showcases how closely mapped our predicted motifs line up with validated TFBS resources.

Using BCrank, these pattern matches can be mapped back to the hg38 reference genome as predicted locations of TFBSs. An example of these predictions is show-cased in Fig 3. These visualizations clearly illustrate the predictive capabilities of the pipeline we developed for matching known and putative motifs within accessible chromatin regions of promoter regions that regulate gene expression.

### Integrated mutation data and interaction of gene regulation mechanisms

**Figure 4:**
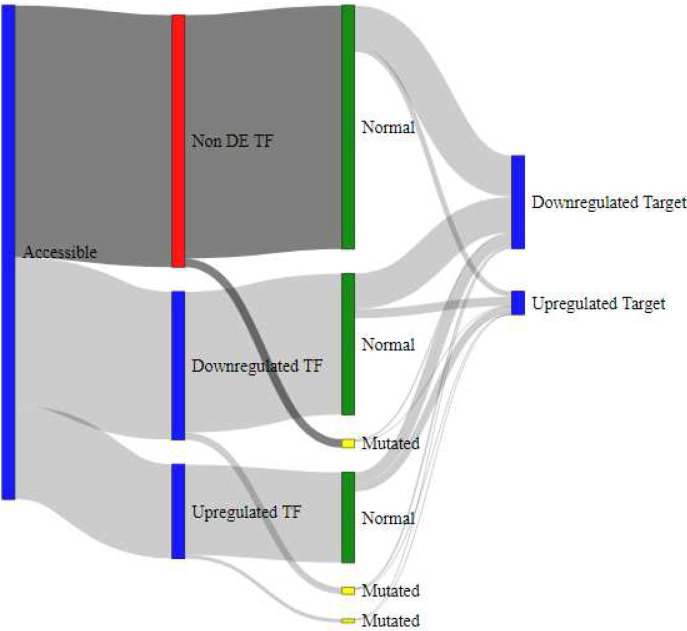
Sankey diagram for subset of TFs with accessible chromatin promoter regions. A subset of this TF list is categorized as differentially and non-differentially expressed (Non DE). This data is further categorized into mutated vs non-mutated TFs. Finally, their target genes are categorized in the right-most column as generally upregulated vs downregulated.

**Figure 5:**
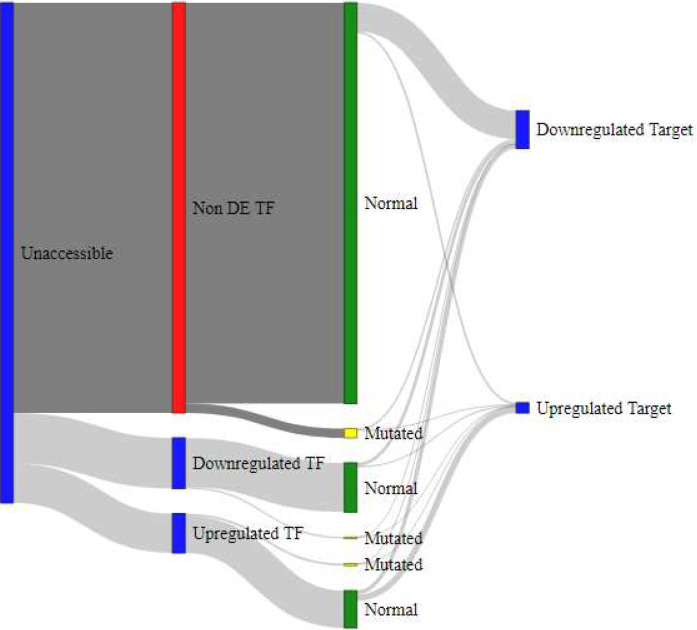
Sankey diagram for subset of TFs with unaccessible chromatin promoter regions. These TFs are more likely be Non DE and produce fewer DE target genes than the accessible TFs in Fig 4.

**Figure 6:**
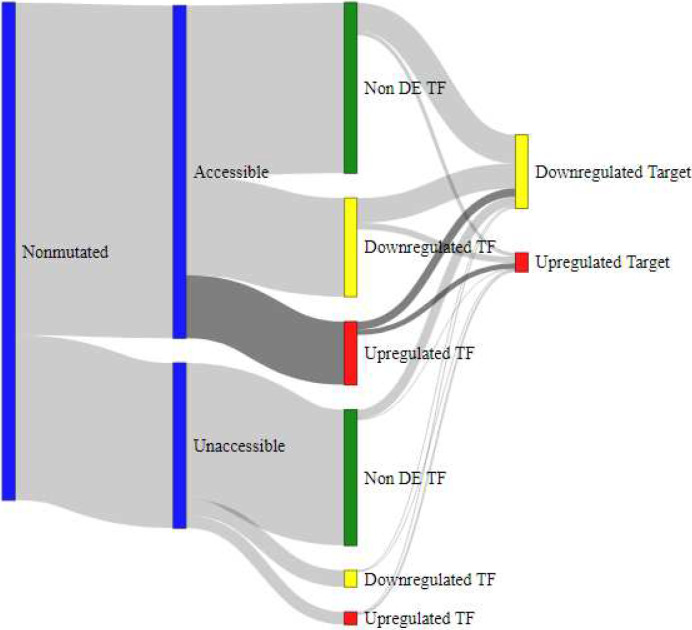
Sankey diagram for subset of TFs without mutations. TFs that are upregulated have a roughly equal likelihood of producing a differentially upregulated target gene or a downregulated target gene.

To model the interaction of gene regulation mechanisms, we employed Sankey diagrams as shown in Fig 4, 5, 6, and 7. These models showcase the epigenetic interactions in a subset of TFs (accessible and nonaccessible, for example). Each TF is classified in terms of their DE pattern, presence or absence of SNP mutations, and DE patterns in the TF’s target genes. Raw data for these measurements is provided as a supplementary table in Additional File 4. SNP mutations lead to structural changes in the mutated TF. In many cases, this behavior prevents TF binding to the promoter DNA sequence, causing downregulation in that TF’s target genes.

Verified target genes for TF regulation were identified using the Harmonizome CHEA Transcription Factor Targets data set [19]. The regulation of target genes was estimated by subtracting the percentage of differentially downregulated target genes in the DDR list from the percentage of differentially upregulated target genes. If the TF target genes were not found in the list of DE genes from DDR, they were not included in the final column of the Sankey diagram. A list of TF genes and their targets used in this analysis is provided as a table in Additional File 5. From these results, we observed that accessible TF genes (Fig 4) were more likely to regulate DE genes than unaccessible TF genes (Fig 5). Furthermore, we observed that accessible TF genes were more likely to produce DE TFs than unaccessible TF genes.

**Figure 7:**
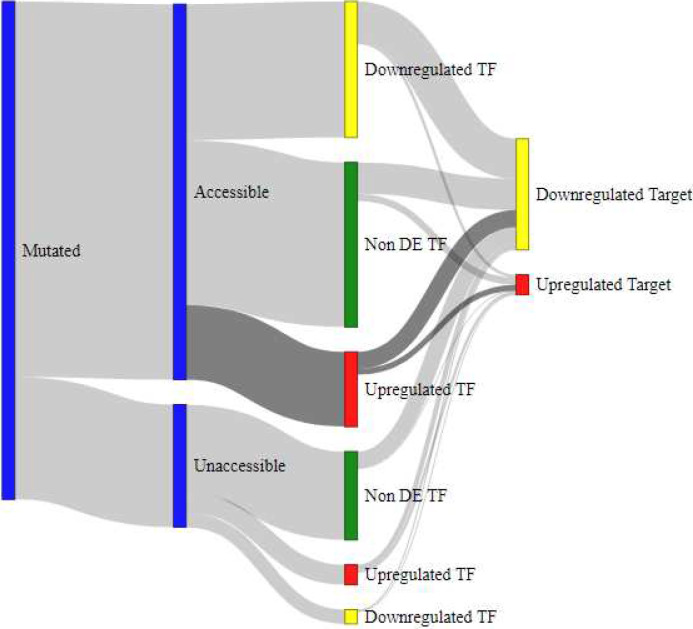
Sankey diagram for subset of TFs with mutations. The subset of TFs that is upregulated and accessible is far more likely to produce a downregulated target when the structural features of the TF are compromised by gene mutation.

Similar diagrams were produced to compare nonmutated TFs (Fig 6) and mutated TFs (Fig 7). In general, mutated genes were more likely to produce differentially downregulated targets. We also observe that upregulated and accessible TFs are more likely to produce downregulated target genes if the TF was mutated, as compared to the nonmutated group. We expected this result because conformational changes to the TF structure prevent it from correctly binding to the TFBS of promoter regions in the TF’s target genes. These expected behaviors help us verify that our pipeline is accurately explaining gene regulation mechanisms and has the potential to be applied to other data sources to understand the origin of disease conditions.

Deviations in the relationship between gene expression of TF accessibility could be explained by gene expression mechanisms unrelated to TFs. For example, gene expression is globally increased in larger cell volumes, rather than on a gene-to-gene basis [20]. RNA polymerase II holoenzyme expression scales with cell volume [20], possibly explaining the mechanism of global gene expression increase, even in genes with downregulated transcription factors. Indeed, experimental models of distributions of gene expression profiles have been improved using exponentially scaling of cell volume [21], as well as other non-TF related gene expression mechanisms including dosage compensation, the exponential rate of mRNA maturation, and the first-order kinetics rate of mRNA decay [21]. Depletion of the cohesin complex and CTCF has been experimentally shown to both upregulate and downregulate gene expression, explained by a variety of mechanisms such as CTCF’s direct binding to the gene’s promoter region [22].

It is also important to recognize that mutations in TFs do not guarantee a loss in gene expression. For example, mutations in the promoter binding site region of the TF are far more likely to compromise the TF’s functionality, as well as regions that complex to other transcription mediators. To further complicate matters, it has been shown that TF families with similar binding site regions are able to substitute mutated TFs, supplementing and reducing the mutation’s impact on overall gene expression [23]. In the future, the overall effectiveness of our tool could greatly be improved by thoroughly investigating and categorizing the impact of mutations on TFs.

### Gene regulation mechanisms in CRC

To better understand gene regulation mechanisms in CRC, we mapped accessible, upregulated TFs to their target genes using the TRRUSTv2 transcription factor database [24]. TRRUSTv2 was chosen because it is a manually curated list that includes metadata for whether the target genes are activated or repressed [24]. The data was further subset to only include TFs that activate differentially upregulated genes or TFs that repress differentially downregulated genes.

As seen in Table 2, a total of 39 transcription factors were identified including CEBPB, the E2F family (E2F1, E2F3, E2F6), the FOX family (FOXA2, FOXL1), MYC, MYCN, and TP73. Transcriptional regulation of BIRC5 via E2F1 has been shown to contribute to the pathogenesis of colorectal cancer [25]. Our data similarly observes that the availability of E2F1 in CRC patients is contributing to the activation of BIRC5, which is also upregulated in CRC.

Notably, several upregulated TFs in CRC are known to interact with apoptosis-regulating genes. E2F1 and MYC both upregulate TP73 (see Table 2). Supporting our observation, TP73 has been shown to block transactivation of TP53, further preventing mechanisms of apoptosis [26]. Additionally, E2F1 and MYCN have repressive interactions with the downregulated TP53 gene.

TP73, BBC3, and PMAIP1 were all found to be differentially upregulated in the CRC data. TP73 functions as a transcription factor that activates BBC3 and PMAIP1, both members of the BCL-2 protein family. Many members of the BCL-2 protein family have been targeted for CRC treatment due to their implications in apoptosis [27]. These observations showcase the potential of our pipeline to easily identify mechanisms of gene regulation in disease. In this case, upregulation of E2F1 and MYC in colon cancer leads to the activation of TP73, further leading to the activation of apoptosis regulators.

## Conclusion

In this paper, we presented a computational approach to quantify gene regulation mechanisms, including TF binding and chromatin accessibility. The advancement of chromatin accessibility technologies like ATAC-seq presents an exciting opportunity to increase our understanding of gene regulation mechanisms in various disease conditions. We validated our theoretical model by showing that there is a statistical relationship between chromatin access and gene expression data in CRC, especially in genes that encode TFs. We believe that this model has great potential to be applied to additional data sets to improve our understanding of the underlying mechanisms behind differential gene expression in other disease conditions, especially in the context of genetic mutations.

## Figures and Tables

**Table 1:** List of data subsets and the respective number of samples in each group.

Data subset	Number of samples

ATAC-seq BAM files	41
Non-duplicated patient IDs	38
Non-duplicated patient IDs with corresponding mutation data	31

**Table 2:** Notable TF-target gene interactions.

TF	Target genes	Interaction Type	Target ED	Pval adjust

E2F1	BIRC5	Activation	1.307	1.46e-16
CEBPB	CXCL8	Activation	1.881	2.12e-20
CEBPB	GDF15	Activation	2.050	2.47e-31
FOXA2	MMP7	Activation	2.056	4.00e-30
FOXA2	ABCA1	Repression	−1.386	3.32e-18
FOXL1	BMP4	Activation	1.381	1.14e-15
E2F1	TP73	Activation	0.355	1.66e-06
E2F1	TP53	Repression	−0.207	3.28e-06
MYC	TP73	Activation	0.355	1.66e-06
MYCN	TP53	Repression	−0.207	3.28e-06
TP73	BBC3	Activation	0.805	7.35e-10
TP73	PMAIP1	Activation	0.978	6.02e-27

List of DE target genes regulated by TFs that were found to be differentially upregulated and accessible in our data set. Target ED is a logarithmic adjustment of fold change for the target gene versus wild type, taken from DDR. Pval adjust is the p-value associated with the Target ED.

## Data Availability

Publicly available data from The Cancer Genome Atlas (TCGA) was used for this study. TCGA Barcode sequences for all used samples can be found in Additional File 1: TCGA Barcodes.
